# An Entropy-Based Condition Monitoring Strategy for the Detection and Classification of Wear Levels in Gearboxes

**DOI:** 10.3390/e25030424

**Published:** 2023-02-26

**Authors:** David A. Elvira-Ortiz, Juan J. Saucedo-Dorantes, Roque A. Osornio-Rios, Rene de J. Romero-Troncoso

**Affiliations:** HSPdigital CA-Mecatronica Engineering Faculty, Autonomous University of Queretaro, San Juan del Rio 76806, Queretaro, Mexico

**Keywords:** condition monitoring, entropy features, gearbox, linear discriminant analysis, statistical features, wear diagnosis

## Abstract

Gears are reliable and robust elements that are found in any power transmission system. However, gears are prone to present incipient faults, such as wear, since they are constantly subjected to contact forces. Due to gears playing a key role in many industrial processes, it is important to develop condition monitoring strategies that ensure the proper functioning of the related power transmission system and the overall components. In this regard, the data on entropy provide relevant information that allow us to identify and quantify the effect of different wear levels in gears. Therefore, in this work, we proposed the use of seven entropy-related features to perform the identification of different wear severities in a gearbox. The novelty of this proposal lies in the use of the entropy features to carry out a high-performance characterization of the available vibration signals that are acquired from experimental tests. The novelty of this proposal lies in the fusion of three different techniques: entropy features, linear discriminant analysis, and artificial neural networks to obtain a machine learning approach for improving the detection of different wear severities in gears compared to other reported methodologies. This situation is achieved due to the high-performance characterization of the available vibration signals that are acquired from experimental tests. Additionally, the entropy features are subjected to a feature space transformation by means of linear discriminant analysis to obtain a 2D representation and, finally, the set of features extracted by linear discriminant analysis are used as inputs of a neural network-based classifier to determine the severity of wear that is present in the gears. The proposed methodology is validated and compared with a conventional statistical approach to show the improvement in the classification.

## 1. Introduction

Gears are important elements in many applications since they are present in every power transmission system [1]. Their popularity is based on the fact that they are robust and reliable pieces that can transmit mechanical power from one component to another but also can achieve modifications in the speed and torque characteristics of the whole system [2]. To perform their work, gears must be in contact with other elements and they are permanently subjected to the action of contact forces that produce constant and gradual wear in the teeth [3]. When the wear level is low, the transmission system can continue working. However, severe wear may result in a broken tooth causing unexpected stops in the processes where it is involved [4]. In this sense, being able to determine the condition of any gear may be helpful to guarantee an optimal power transmission and an accurate speed ratio. Therefore, there exists a need for the development of strategies and methodologies that can evaluate different severities in the wear level to prevent catastrophic failures [5].

Due to the importance of detecting failures on the gearboxes at early stages, several techniques have been already proposed to address this issue. All of the so far reported techniques use different physical signals in order to obtain information regarding the operating conditions of the system. In this sense, electrical signals (voltage and current) [6], vibrations [7], temperature [8], and acoustic emissions [9], are common magnitudes that allow us to determine the existence of fault conditions in many electromechanical systems, including gearboxes. As the gearboxes are commonly connected to an electric motor, the motor current signature analysis (MCSA) technique has been widely used for the detection of faults in gearboxes. These techniques use frequency domain transforms as the fast Fourier transform (FFT) [10,11], or time–frequency transformation as wavelets [12], multiple signal classification (MUSIC) [13], and empirical mode decomposition (EMD) [14] to extract behavioral patterns that change from one operating condition to another. Additionally, the information obtained from the domain transforms usually works along with some artificial intelligence techniques, such as artificial neural networks (ANNs) [15] and fuzzy systems [16] that work as classifiers to perform automatic detection of the type or severity of fault that is present in the gearbox. MCSA is a simple and effective solution for identifying tooth wear in gears and other faults; yet, the current measurement is not carried out directly in the gearbox, hindering the detection of anomalies at early stages. In this sense, vibration measurement has proven to be a robust and reliable alternative. Moreover, since the vibration signals can be directly acquired from the gearbox, this technique results more useful for dealing with incipient fault conditions [17]. Therefore, an important number of works using vibration analysis have been developed, following a transform-based scheme, similar to the one used with the stator current signals [18,19,20,21]. Even though vibration analysis can improve the accuracy in the detection of wear condition in gears, the transform-based techniques still have some drawbacks. For instance, most of the time–frequency transformations require a high computational burden and their implementation is slow and not feasible to be implemented for online monitoring systems. Additionally, some techniques, such as wavelet and EMD, suffer from mode mixing, a situation that may lead to erroneous results.

To deal with some of the disadvantages associated with the transform-based approaches, some works propose the extraction of features directly from the time domain vibration signal. In this regard, the statistical features are the most widely used [22,23,24]. In these feature-extraction approaches, the statistical features are used to identify the different patterns from one operating condition to another and this information is used as input for a classifier in order to identify the condition of the gearbox. Such implementation usually reduces the computational effort as well as the execution time, and the accuracy of the results is good. Notwithstanding, the number of extracted features can be high and discriminate between those that are relevant and those that do not a great impact. Therefore, to reduce the number of features without losing important information, some machine learning (ML) techniques, such as linear discriminant analysis (LDA) [25] or principal component analysis (PCA) [26], are implemented. These last two techniques allow for performing feature reduction as well as a classification at the same stage. Nevertheless, they can be used along with another classification scheme to improve the accuracy of the results. The main issue regarding the use of statistical features is that they can lead to misclassifications in noisy environments. In recent years, it has been demonstrated that an alternative to statistical features is the use of entropy features (EFs). EFs are particularly good for identifying dynamic behaviors in nonlinear signals by measuring the degree of complexity of the data [27]. This situation makes EFs important tools for identifying the fault-related information merged into the vibration signals. There is a wide variety of EFs such as the energy entropy, the permutation entropy, the sample entropy, the approximate entropy, and the fuzzy entropy, just to mention a few; each one of these parameters provides different information that can be useful for the detection of a variety of faults in gearboxes such as wear, broken teeth, eccentricity, and imbalance, among others [28,29]. Moreover, it has been demonstrated that EFs are suitable for automatically detecting different faults in gearboxes if they are considered along with ML techniques and classification algorithms, such as support vector machines (SVMs) [30] and k-nearest neighbors (KNNs) [31]; and more recently with deep learning (DL) techniques, such as convolutional neural networks (CNNs) [32] and autoencoders [33]. However, despite DL techniques leading to achieving advantageous results, their implementation is associated with a high computational burden because of the processing of images (i.e., CNNs), and a priori knowledge is required to set specific hyperparameters (i.e., autoencoders). Although EFs allow for increasing the effectiveness in the detection of faults in gears, the right selection of a specific feature is crucial to ensure accurate results. Moreover, there is still a need of developing strategies for detecting different wear severities in gears since most of the so far reported works only deal with cracks and broken teeth.

The present work proposes a methodology that uses a set of seven different entropy features calculated over the vibration signals of a gearbox in two different axes with the purpose of detecting four distinct wear severities in gears. To reduce the amount of information, the LDA technique is applied to the set of EFs, allowing a 2D representation of the different operating conditions to be obtained. The reduced set of features coming from the LDA is sent to an ANN classifier that delivers as output the level of wear level in the gears. Experimentation is performed using real signals coming from a kinematic chain formed by a DC generator, and an induction motor coupled between them by means of a gearbox. The proposed approach is compared with a methodology that uses statistical features and the results show that the EF provides better performance and increases the percentage of accuracy in the classification of the wear severity. Although the techniques presented in this work have been separately used in condition monitoring tasks, it has not been explored how they work together. Therefore, the main contribution of this work consists of the fusion of the entropy features, the LDA technique, and ANNs to obtain a machine learning approach that can detect and classify different levels of wear in gearboxes. Moreover, the proposed methodology considers the identification of incipient and gradual faults that are not typically reported in the literature. The proposed approach aims to improve the results delivered by the so far reported techniques showing that the fusion of these techniques results in a better classification scheme for the early detection of wear in gearboxes, preventing unexpected stops and financial losses.

## 2. Materials and Methods

In this section, we describe the theoretical aspects that have to be considered for applying the proposed diagnosis methodology. Section 2.1 describes the theory of Shannon entropy and variants, while Section 2.2 depicts the theoretical formulation of the supervised LDA feature reduction technique.

### 2.1. Shannon Entropy

The concept of entropy appears in many areas of science and technology, but entropy is usually defined as the degree of information and/or misinformation that is available for a system. On the other hand, Shannon entropy is an important concept in information theory with multiple applications in computer science, telecommunications, and deep learning, among others. It refers to the amount of information that an event or probability distribution provides [29]. Thus, in condition monitoring strategies, Shannon entropy may be used as a metric leading to model trends and dynamic changes that are produced by undesirable operating conditions.

In this regard, Shannon entropy is applied to a discrete variable X which is composed of n values of $x_{i}$, as (1) depicts, and the corresponding Shannon entropy is estimated by (2):

(1) $$X=\left\{x_{1},\;x_{2},\;x_{3},\ldots ,x_{n}\right\},\;\;\;\;\;\;i=1,\;2,\;3,\ldots ,\;n$$

(2) $$H\left(X\right)=-\sum_{i=1}^{n}p\left(x_{i}\right)\log\left(p\left(x_{i}\right)\right).$$

where p is considered as the ratio between each $x_{i}$ and the total number of data and/or the probability of X for a value $x_{i}$ and n is the available data length.

#### Variants of Shannon Entropy

The formulation of Shannon entropy has led to different variants. Some of the most important are energy entropy (EnE), permutation entropy (PeE), rényi entropy (ReE), sample entropy (SaE), approximate entropy (ApE), and fuzzy entropy (FuE), among others [34,35]. In this regard, EnE is commonly used to measure irregularities in data series and for its understanding. It can be considered a set of intrinsic mode functions (IMFs) as the functions to describe the conditions of a rotating machine; thus, if there are p available IMFs, the equivalent EnE is computed by applying Equations (3)–(5) [36]; where the energy of each IMF with a length of q is first computed in Equation (3), the total energy for the p IMFs is carried out in Equation (4) and, based on these two previous equations, estimated EnE by Equation (5):

(3) $$E_{i}=\sum_{j=1}^{q}{\left|c_{ij}\right|}^{2}$$

(4) $$E=\sum_{j=1}^{p}E_{i}$$

(5) $$EnE=-\sum_{i=1}^{n}p_{i}\log\left(p_{i}\right)$$

where $p_{i}=E_{i}/E$ is the ratio of the probability of the energy for each IMF regarding the total energy entropy.

PeE is a proposed value that can quantify the complexity of disorder in a data series and it also has the capability for modeling dynamic features during the monitoring of rotatory machines [37]. In general, the association of the probability distribution ∏ (with $\pi_{i}$ elements) are the permutation patterns that are related frequencies. Thus, if i=1,…,D!, PeE is defined by Equation (6):

(6) $$PeE=-\sum_{i=1}^{D!}\pi_{i}\ln\pi_{i}$$

ReE has been proposed as a quantitative metric to compute irregularities, unpredictability, and randomness of a data series [38], and it can be estimated by applying Equation (7), where $p_{i}$ is the ratio or probability of each value $x_{i}$ and the order is depicted by α. Indeed, ReE has two advantages. The first one is that it is not sensible to change regardless of the density function, and the second is that it also offers to modify the data series by a given scaling factor due to the additive factor.

(7) $$ReE=-\frac{\alpha }{1-\alpha }\sum \log_{2}p_{i}^{\alpha }$$

Similarly, SaE is a measure used to estimate the complexity of data series, precisely. Such complexity is quantified from the viewpoint of the similarity coefficient r and m as the embedding dimension; hence, a smaller complexity in the data series is depicted by a small value in ReE. Meanwhile, a large value of ReE is associated with complex data series [39]. Therefore, ReE is estimated by Equation (8), where $B^{m}$ represents the mean of a pattern.

(8) $$SaE=-\ln\left(\frac{B^{m+1}\left(r\right)}{B^{m}\left(r\right)}\right)$$

On the other side, ApE is usually estimated to obtain a metric that describes irregularities and unpredictabilities in data series and it can be estimated as Equation (9) describes, where r and m are the tolerance and pattern length, respectively [40]. The computation of ApE leads to obtaining significant advantages. First, for a small data series, it is possible to achieve stable prediction results, second, it can be applied to random and certain signals, and third, it can deal with the interference feature. Nevertheless, although advantages are achieved, its estimation is considered a low-efficiency process.

(9) $$ApE=\phi^{m}\left(r\right)-\phi^{m+1}\left(r\right)$$

Finally, FuE can be used to achieve better separability between the boundaries of two classes, as well as to measure the uncertainty of data series [41]. FuE is estimated by Equation (10), where r represents the similarity coefficient and n is the gradient.

(10) $$FuE=\ln\varphi^{m}\left(n,r\right)-\ln\varphi^{m+1}\left(n,r\right)$$

### 2.2. Dimensionality Reduction

Condition monitoring strategies are commonly supported by ML techniques in order to accomplish a specific goal. However, the use of dimensionality reduction techniques is preferred since they allow, first, the understanding of the data distribution, and second, obtaining the representation of an original feature space into a 2*d* and/or 3*d* space [42,43]. In this sense, principal component analysis (PCA) is an unsupervised dimensionality reduction technique that can be used for interpretability purposes. Specifically, PCA is a technique based on data variance, which is retained as much as possible during the reduction procedure. In condition monitoring strategies, PCA has been used to reduce the dimension of high-dimensional feature space and subsequently for interpreting the distribution of original feature space [44]. On the other hand, linear discriminant analysis (LDA) is a supervised dimensionality reduction technique that is ideal for being applied in multi-class classification problems. The objective of the LDA technique resides in maximizing as much as possible the linear separation between two or *C* classes. Therefore, LDA has advantages when the condition monitoring of different faults is intended to be performed [45].

LDA can be performed by three consecutive steps for a given and original feature matrix $X=\left\{x_{1},\;x_{2},\;x_{3},\ldots ,x_{N}\right\}$, where each $x_{i}$ belongs to each *i-th* sample of a specific pattern or feature, and each one of these samples is denoted by *M* features; that is, the feature matrix X has an *M*-dimensional representation with *N* consecutive samples ($x_{i}\;\in \;{\mathbb{R}}^{M}$). Consequently, in the first step, it is necessary to compute the separability between *C* considered classes. Such separability can be quantified in terms of the distances between the means of the classes by estimating the between-class matrix or the between-class variance, as depicted by Equation (11), where $\left(m_{i}-m\right)$ denotes the distance between two different classes, m is the overall mean for all considered classes, $m_{i}$ is the mean for the *i-th* class, and $N_{i}$ the number of samples for the *i-th* class.

(11) $$S_{B}=\sum_{i=1}^{C}N_{i}\left(m_{i}-m\right){\left(m_{i}-m\right)}^{T}$$

Subsequently, in the second step, we calculated the distance between each $m_{i}$ and its corresponding samples of the *i-th* class. This step is also known as the estimation of the within-class matrix or the within-class variance that is carried out by following Equation (12).

(12) $$S_{W}=\sum_{i=1}^{C}\left(x-m_{i}\right){\left(x-m_{i}\right)}^{T}$$

Once the between-class matrix ($S_{B}$) and within-class matrix ($S_{W}$) are estimated, the transformation matrix W is computed by means of Equation (13), which is also known as the criterion of Fisher. Then, Equation (13) is rewritten as Equation (14) and the solution of W is found by solving the general eigenvalue problem, where λ contains the corresponding eigenvalues of W and $W=S_{w}^{-1}S_{B}$ if $S_{W}$ is a nonsingular matrix.

(13) $$arg\;\underset{W}{max}\frac{W^{T}S_{B}W}{W^{T}S_{W}W}$$

(14) $$S_{W}W=\lambda S_{B}W$$

Finally, the new set of features Y is extracted and projected into a lower dimension by multiplying the original feature space X with the transformation matrix W, as in Equation (15).

(15) Y=XW

## 3. Methodology

**Figure 1:**
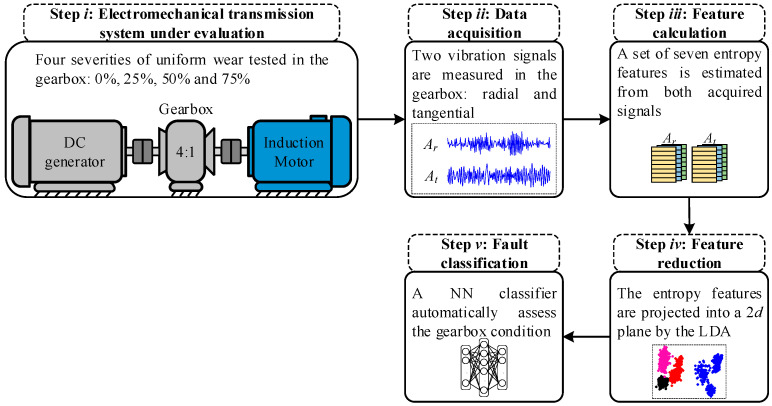
Flow chart of the proposed method for assessing four different severities of uniform wear in an electromechanical transmission system under different operating frequencies.

The proposed method for assessing different severities of uniform wear in an electromechanical transmission system consists of five main stages, as Figure 1 depicts: the electromechanical transmission system under evaluation, the data acquisition, the feature calculation, the feature reduction, and the fault classification.

*Step i*: Firstly, in the electromechanical transmission system under evaluation, four different conditions are experimentally tested in a 4:1 ratio gearbox. The assessed conditions belong to three severities of uniform wear (25%, 50%, and 75%) and healthy or unworn gear (0%). These severities are iteratively tested in the gearbox and each one of them is evaluated under different supply frequencies that produce different rotating speeds. The supply frequencies are 5 Hz, 15 Hz, 50 Hz, and 60 Hz.

*Step ii*: In the data acquisition stage, two vibration signals are acquired from the perpendicular plane of the gearbox rotating axis. Indeed, it has been proven that the mechanical vibrations produced along the radial ($A_{r}$) and tangential ($A_{t}$) axes provide significant information regarding the condition of rotating machinery [46]. Thus, for each condition of the gearbox and for each tested supply frequency, both vibration signals are measured through the accelerometer sensor and recorder in a personal computer for posterior analysis. The vibrations signals are continuously monitored during 100 s of the operations of the electromechanical transmission system.

*Step iii*: In the third stage, the feature calculation is performed in order to obtain the characterization of the acquired vibration signals. In this regard, the characterization process is based on the estimation of a meaningful set of seven entropy features. The set of features is composed of Shannon entropy (ShE), energy entropy (EnE), permutation entropy (PeE), rényi entropy (ReE), sample entropy (SaE), approximate entropy (ApE), and fuzzy entropy (FuE). The corresponding formulation of these entropy features has been presented in Section 2.1. Therefore, to carry out the feature calculation, both acquired signals are individually segmented in identical parts of one second. Then, each segment is computed with a proposed set of seven entropy features and as a result, it obtains a characteristic feature matrix $EF\;\in \;{\mathbb{R}}^{EF}$ composed of EF=14 entropy features (7 per signal) and 100 consecutive samples. Due to different conditions of uniform wear are tested under different supply frequencies, a characteristic feature matrix is estimated for each one of the experiments.

*Step iv*: For feature reduction, one of the well-known ML techniques is used to achieve a dimensionality reduction. In that way, the original characteristic feature matrices ($EF\;\in \;{\mathbb{R}}^{EF}$) are subject to a space transformation by means of LDA, where their original dimension is reduced to a lower one. A new set of features are extracted through this dimensionality reduction procedure and these extracted features are estimated as the linear combination (in different weights) of the original features. For this proposal, the extracted features are projected into a 2D plane allowing the visual representation of all evaluated conditions. Additionally, the implementation of this reduction procedure leads to facilitating the classification task for a specific classification algorithm.

**Figure 2:**
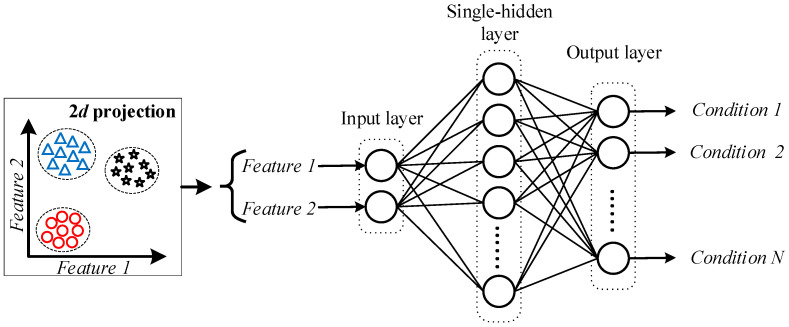
Structure of the proposed NN-based classifier used to perform the automatic diagnosis of different gearbox fault severities.

*Step v*: In the fault classification, the automatic detection of uniform wear is performed by a proposed neural network (NN) classifier. The proposed NN classifier is a single structure that only consists of three main layers, where, in the input, layers defined two neurons since in the previous stage they are extracted two new features that are projected into a 2D space. Then, the hidden layer is defined as a single-hidden layer with ten neurons as recommended in the literature [47], and in the output, the layer is defined by a number of neurons equal to the assessed conditions; that is, the output layer has four neurons. The training and testing of the proposed NN structure are accomplished under a five-fold cross-validation scheme to obtain statistically significant results. Moreover, the NN structure is trained under a backpropagation algorithm during 100 epochs. Finally, the use of the NN structure also allows the modeling of the decision regions that can be used to analyze and estimate the posterior probability for those misclassification samples. Figure 2 shows a detailed description of the proposed structure used in the NN-based classifier. The resulting feature space obtained by LDA (Feature 1 and Feature 2) is considered to be evaluated in the input layer.

## 4. Experimental Setup

**Figure 3:**
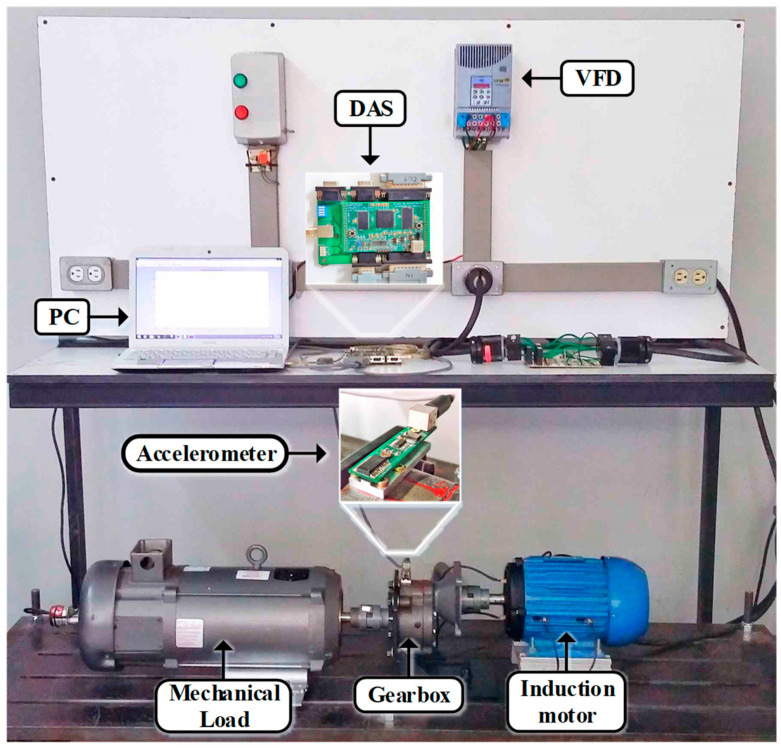
Test bench for the wear severity evaluation.

**Figure 4:**
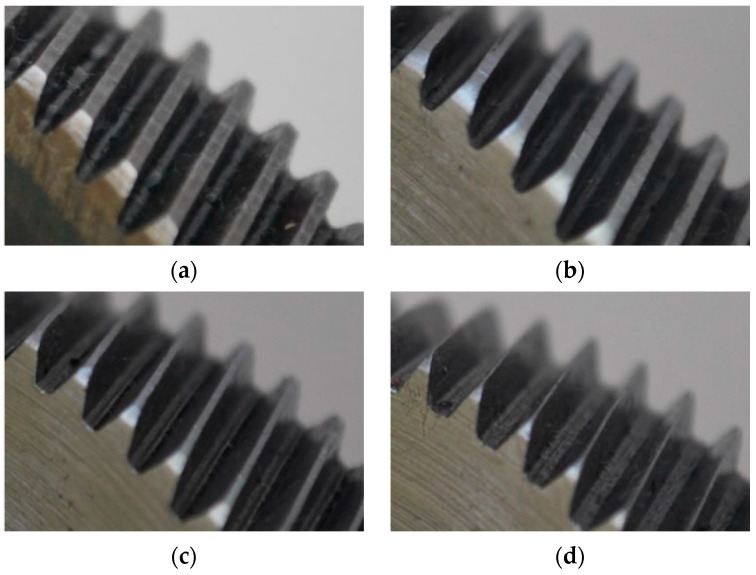
Set of gears used during the experiments for analyzing its operating condition: (**a**) healthy gear and gears with (**b**) 25%, (**c**) 50%, and (**d**) 75% of uniform wear.

The proposed methodology is validated using the test bench presented in Figure 3. This test bench uses a 1492 W three-phase induction motor (IM), model WEG 00236ET3E145T-W22, which operates at a rated voltage of 220 V. This motor is mechanically coupled to a gearbox through a rigid coupling. The gearbox is a BALDOR GCF4X01AA with a single-stage 4:1 ratio. To test the proposed methodology for the detection of different wear severities, four different gears are mounted in this gearbox. First, a healthy; then, a gear that was carefully manufactured to present 25% wear; next, a gear with 50% wear; and last, a gear that presents 75% wear. The different severities of wear are artificially induced by means of a manufacturing process in which all gear teeth are uniformly worm in different percentages (25%, 50%, and 75%) with the aim of reducing the top land width in the whole teeth, as shown in Figure 4. Additionally, a DC generator model, BALDOR CDP3604, is attached to the other side of the gearbox to work as a mechanical load for the system. To control the startup and the operating frequency of the IM, we used a variable frequency driver (VFD) from the WEG model CFW08. The vibration measurement is performed using a triaxial accelerometer model, LIS3L02AS4, that is mounted on the top of the gearbox, as shown in Figure 3. The data delivered by the accelerometer is acquired using a proprietary data acquisition system (DAS) that uses a field programmable gate array (FPGA) as the main technology. The DAS implements a 3 kHz sampling frequency and is able to simultaneously acquire the data from the three axes of the sensor. All data are stored in a personal computer to be processed later. Every test has a 100 s duration; yet, they are divided into windows with 100 samples in each one. As aforementioned, every wear condition is tested under four different operating frequencies (5 Hz, 15 Hz, 50 Hz, and 60 Hz). Therefore, every test is formed for a total of 400 samples.

## 5. Results and Discussion

The aforementioned methodology is implemented using the Matlab© software in order to demonstrate its effectiveness in the detection and classification of the four operating conditions of the gearbox (0%, 25%, 50%, and 75% of uniform wear). Moreover, to show that there exists an improvement, the proposed EF-based methodology is compared to a conventional implementation using statistical features. The only difference between the proposed methodology and the statistical approach is the feature set that is calculated at step *iii*, where the EF is replaced for the statistical features presented in Table 1.

**Figure 5:**
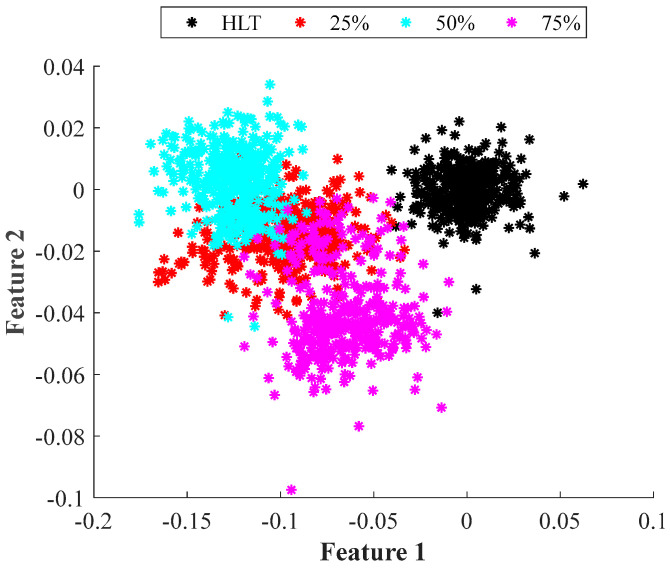
Achieved 2D representation performed by applying supervised LDA to the classical set of statistical features for the four severities of wear tested in the gearbox under different operating frequencies.

In Figure 5, we present the result of applying LDA over the set of statistical features summarized in Table 1. It is observed in four different clusters: one per each wear condition. In Figure 5, it is noticeable that the healthy condition (black cluster) is separated from the rest of the wear severities; nevertheless, there is a severe overlap among the other three conditions. In fact, the 50% wear condition (cyan cluster) and the 75% wear condition (magenta cluster) seem to cover the left half and the right half, respectively, of the 25% wear condition (red cluster). Thus, it can be inferred that if the two features delivered by LDA are used as inputs of any classifier, several misclassifications would appear among the 25%, 50%, and 70% wear severities.

**Figure 6:**
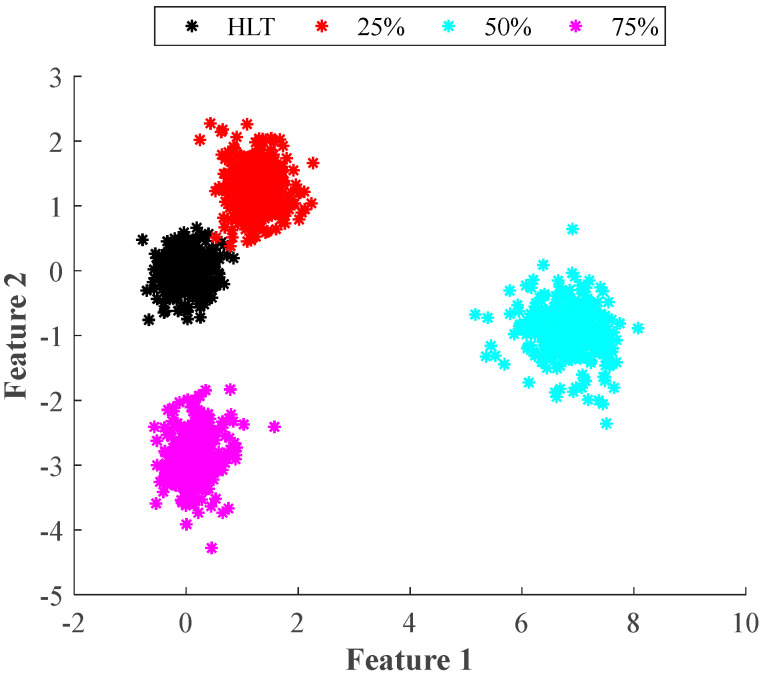
2D visual representation obtained by the supervised LDA technique applied to the original entropy feature matrices for the four severities of wear tested in the gearbox under different operating frequencies.

On the other hand, when the statistical features are replaced by the proposed EF, the LDA technique delivers the groups shown in Figure 6. Now, the separation among all the classes (wear severities) has increased. Only the healthy condition (black cluster) and the 25% wear condition (red cluster) remain close to each other. This is an expected situation since the 25% wear severity is an incipient fault state that presents a behavior pattern very similar to the healthy condition. However, LDA is able to differentiate between these two similar conditions. In this case, due to the clear separation among classes, it is expected an improvement in the classification task when the two features delivered by LDA are used as inputs of any classifier. 

**Figure 7:**
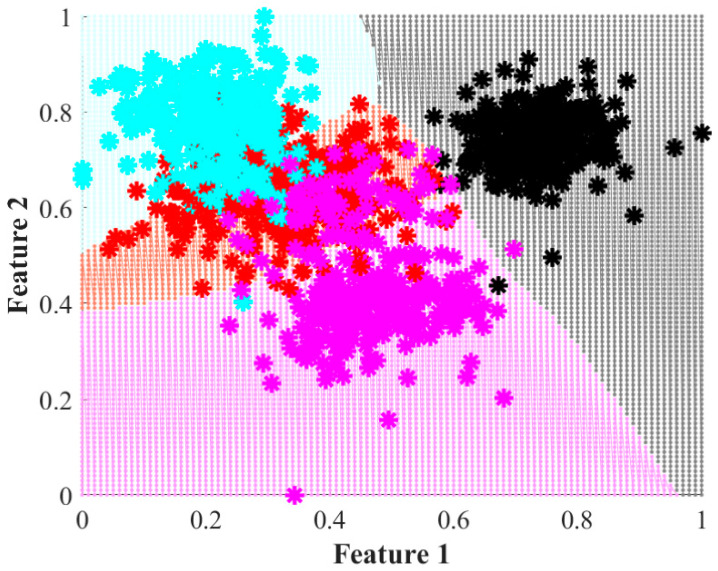
Decision regions achieved during the training of the proposed NN-based classifier when analyzing the extracted features by LDA over the statistical features. The decision conditions are modeled but overlapping between them appears in black, red, cyan, and magenta, which represent the HLT, 25%, 50%, and 75% conditions.

To carry out the classification, a simple perceptron NN is implemented considering two neurons at the input layer (one per each feature delivered by LDA), a hidden layer of ten neurons, and an output layer of four neurons (one per each wear severity). As has been previously stated, every operating condition has tests composed for a total of 400 samples. Since there are four different operating conditions, a total of 400 samples are considered in the classification stage. From the total, 1280 samples are used for the training process of the NN and the remaining 320 samples are left for the validation task. Figure 7 presents a visual representation of the decision regions obtained by the NN during the training process when the inputs are the features obtained by LDA with the statistical approach. The four regions can be clearly identified. Notwithstanding, it can be observed that many of the cases are misclassified, especially those that fall into one of the conditions different from the healthy state.

**Figure 8:**
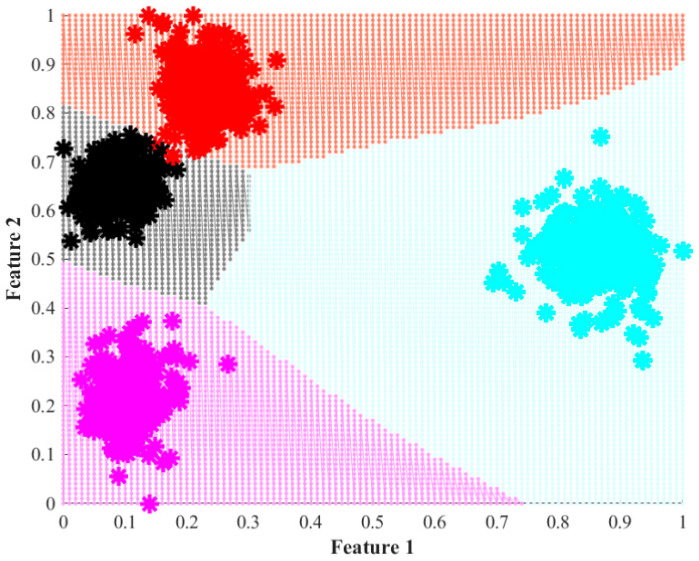
Decision regions achieved during the training of the proposed NN-based classifier when the extracted features by LDA over the entropy features are analyzed. Each assessed condition is modeled by its decision region in black, red, cyan, and magenta, which represent the HLT, 25%, 50%, and 75% conditions.

Figure 8 presents the decision regions that are obtained when the classification task is performed using the features obtained with LDA and the EF. In this case, it is clear that there is an improvement in the classification since most of the cases fall into the correct classification region. Again, it is observed that some of the cases that present a 25% wear severity fall into the region tagged as a healthy state; yet, the amount of errors made by the NN classifier is much lower using the EF than using the statistical features. Moreover, when the statistical features are used, an overlap appears among three of the four conditions, whereas in the case of the EF, the overlap is minimum and it is only observable between two conditions.

Accordingly, Table 2 and Table 3 present the confusion matrices obtained by the classifier using LDA over the statistical features and the EF, respectively. They presented the results of both the training and the validation processes. For the case of the statistical approach, an overall classification ratio of 86.6% is achieved during the training and 85.3% for the validation. Here, it is worth noticing that for the healthy case, the classifier makes only a few mistakes. This situation corresponds with Figure 5 and Figure 7, where it is observed that the healthy case is separated from the other three wear conditions. Additionally, it is observed in Table 2 that most of the classification errors occur in the 25%, 50%, and 75% wear conditions, a situation that can be easily explained by the overlap among classes that is visible in Figure 5 and Figure 7. For its part, the classifier that uses the features obtained with LDA and the EF achieves global classification ratios of about 99.7% for the training and validation. This represents an improvement of almost 13% in the identification of wear in the gears even at early stages. It must be mentioned that in this case and according to the data presented in Table 3, misclassifications appear between the healthy and the 25% wear conditions. This situation agrees with the presented in Figure 5 and Figure 7 where there appears to be little overlap between these two conditions, and the remaining conditions are widely separated from each other.

Thus, it can be affirmed that the use of the EF increases the reliability of the process for the identification of wear conditions in gears. The combined LDA and EF methodology can accurately detect different severities of wear even at early stages making it a robust and reliable tool that can be useful to prevent unexpected stops and financial losses at industrial facilities.

Additionally, the fault assessment through the analysis of fault-related frequency components by means of FFT is performed in order to highlight the feasibility of the proposed method in contrast with classical approaches. Thus, to achieve the fault diagnosis, we estimated the meshing frequency ($f_{m}=N\cdot f_{r}$), which is also known as the gearbox fault-related frequency. In this regard, it is mandatory to know the teeth number (*N*) of the gear under study, as well as its rotating frequency (*fr*). Additionally, for gearboxes in healthy conditions, the vibration spectra usually present the input and output shaft frequencies, the $f_{m}$ with sideband frequencies ($f_{sideband}=f_{m}\pm f_{r}$) around the $f_{m}$, and its corresponding harmonics. Certainly, the gearbox under study has a pair of gears where the drive gear has 18 teeth and the driven has 72 teeth. Indeed, the driven gear is that gear where different uniform severities of wear have been induced. In this sense, in Table 4, we summarized the estimated frequencies of interest to assess the gearbox condition. Table 4 provides information related to the rotational speed of the induction motor, the rotating frequencies of the drive ($f_{r\_drive}$), and the driven gear ($f_{r\_driven}$), as well as the first and second harmonics linked to the meshing frequency ($f_{m}$ and $2f_{m}$).

**Figure 9:**
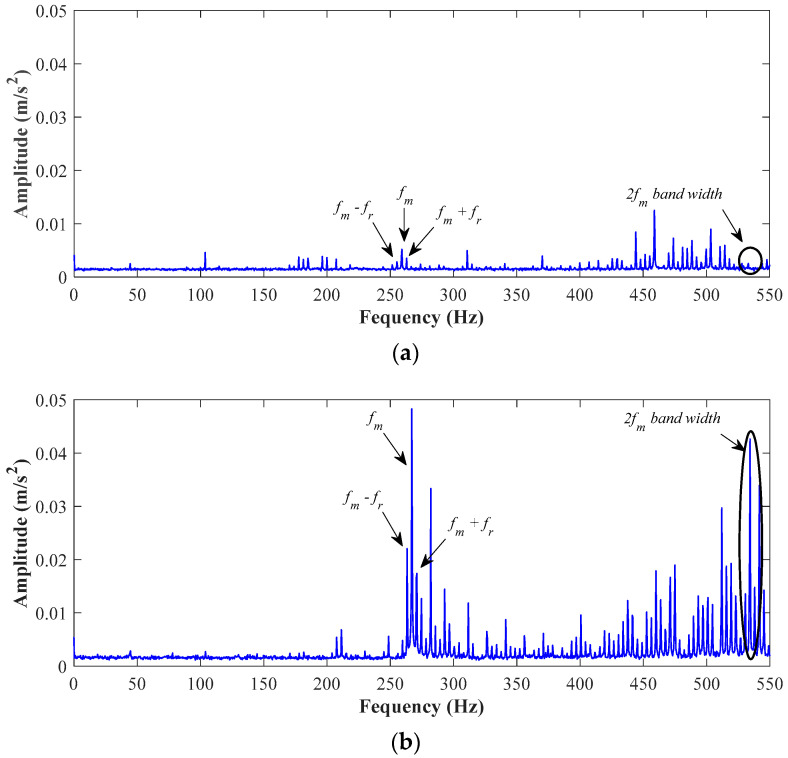
Vibration spectra of the experiment with VFD set to 15 Hz for (**a**) the healthy condition and (**b**) the condition of 50% of uniform wear.

Subsequently, the vibration spectra are estimated through FFT to analyze the different conditions. Thus, Figure 9a,b show the spectra that belongs to the HLT condition and the condition with 50% of uniform wear, respectively, when the VFD is set to 15 Hz. In both spectra, it is possible to detect significant frequencies, such as the mesh frequency ($f_{m}=267.3\;\mathrm{Hz}$) and its corresponding second harmonic ($2f_{m}=534.7\;\mathrm{Hz}$). In Figure 9a,b, it is possible to notice that in the spectrum for the HLT condition, the related frequencies are present with a low amplitude level, and the appearance of unexpected vibration components is present in the spectrum. Specifically around the second harmonic in the $2f_{m}$, the lateral sidebands are full of additional frequency components. On the other hand, in the spectrum for the condition of 50% of uniform wear, the frequency amplitude increases significantly, and this increase is directly associated with an improper working condition on the gearbox.

Finally, with the aim of comparing the performance achieved by the proposed method in comparison with classical approaches, the proposed NN-based classifier is used to evaluate different sets of features. In this regard, the NN classifier is individually evaluated by (*i*) the set of the EF (without any reduction process), (*ii*) the set of statistical features (without any reduction process), and (*iii*) the frequency spectrum estimated by FFT. Hence, the achieved classification ratios obtained during the training and test under a 5-fold cross-validation scheme with a backpropagation algorithm and 100 epochs are summarized in Table 5. As Table 5 describes, the proposed method based on the use of the EF, the LDA technique, and the NN classifier has superiority over classical approaches.

## 6. Conclusions

Due to the importance of gears in power transmission systems, it is necessary to count on reliable methodologies for the detection of wear in the gears at early stages with the aim of taking preventive maintenance to avoid catastrophic faults that result in losses. In this sense, this work proposes an approach whose main contribution is the fusion of EF, LDA, and ANN to obtain a machine learning methodology that improves the detection and classification of wear severities in gearboxes. The conventional methodologies based on statistical features lead to misclassification errors when dealing with gradual wear severities. Therefore, a good alternative to the use of statistical features is the use of entropy features. Entropy features allow us to perform a detailed track of the behavior of nonlinear signals and they are able to identify differences among operating conditions even when their behaviors are similar. In this sense, the use of entropy features increases the reliability of the techniques for the early detection of wear levels in gearboxes. Moreover, the proper selection of the features to be used for tracking every operating condition may result challenging. Yet, the LDA technique allows to discern among the features and reduces the dimensionality of the data set to simplify the interpretation of the operating conditions. The proposed methodology proved to be effective for the detection of wear severities in gearboxes even at different operating frequencies, making it a desirable tool for preventive maintenance tasks that help to avoid financial losses and safety hazards in industrial facilities.

## Figures and Tables

**Table 1:** The proposed set of statistical features for the comparison with the EF-based methodology where x(i) is the i-th sample for i = 1, 2, …, N and N is the number of points for each acquired signal.

Statistical Time Domain Feature	Mathematical Equation
Maximum value	$T_{1}=max\left(x\right)$
Root mean square	$T_{2}=\sqrt{\frac{1}{N}\cdot \sum_{i=1}^{N}{\left(x_{i}\right)}^{2}}$
Standard deviation	$T_{3}=\sqrt{\frac{1}{N}\cdot \sum_{i=1}^{N}{\left(x_{i}-T_{1}\right)}^{2}}$
RMS shape factor	$T_{4}=\frac{T_{2}}{\frac{1}{N}\cdot \sum_{i=1}^{N}\left|x_{i}\right|}$
Crest factor	$T_{5}=\frac{T_{1}}{T_{2}}$

**Table 2:** Confusion matrix achieved during the training and test of the proposed NN-based classifier applied to the extracted features by LDA over the statistical features.

		Actual							
		Training				Validation			
	Condition	HLT	25%	50%	75%	HLT	25%	50%	75%
Estimation	HLT	**318**	0	0	2	**80**	1	0	0
	25%	1	**272**	50	67	0	**56**	9	44
	50%	0	38	**268**	0	0	16	**71**	0
	75%	1	10	2	**251**	0	7	0	**66**

**Table 3:** The confusion matrix was obtained by applying the proposed NN-based classifier to the extracted features by LDA over the proposed entropy features.

		Actual							
		Training				Validation			
	Condition	HLT	25%	50%	75%	HLT	25%	50%	75%
Estimation	HLT	**319**	0	0	0	**80**	1	0	0
	25%	1	**320**	0	0	0	**79**	0	0
	50%	0	0	**320**	0	0	0	**80**	0
	75%	0	0	0	**320**	0	0	0	**80**

**Table 4:** Estimated frequencies of interest to assess the gearbox condition.

Rotational Speed (rpm)	Frequencies of Interest (Hz)			
	$f_{m}$	$2f_{m}$	$f_{r\_drive}$	$f_{r\_driven}$
293.4	88.02	176.04	4.89	1.22
889.28	266.79	533.58	14.82	3.70
2984.4	895.32	1790.65	49.74	12.43

**Table 5:** Performance was achieved by considering several approaches during the training and testing the proposed NN-based classification.

		Training	Test
Other approaches	EF + NN	74.7%	75.0%
	SF + NN	66.2%	66.6%
	FFT + NN	60.5%	61.7%
	SF + LDA + NN	86.6%	85.3%
**Proposed method**	**EF + LDA + NN**	**99.7%**	**99.7%**

## Data Availability

Data are available upon request.
